# Etiology of Hearing Impairment in Children and Adolescents of a Reference Center APADA in the city of Salvador, state of Bahia

**DOI:** 10.1016/S1808-8694(15)30031-8

**Published:** 2015-10-19

**Authors:** Luzia Poliana Anjos da Silva, Fernanda Queiros, Isabela Lima

**Affiliations:** a Assistant Speech and Hearing Therapist at APADA/ BA (Association of Parents and Friends of the Hearing Challenged of the State of Bahia); Master's student of Medicine and Health. Emphasis: Neurosciences – Medical School at UFBA, Resident physician in Neonatology – Ministry of Health / UNEB; b Master's student of Medicine and Health. Emphasis: Neurosciences - Medical School at UFBA.; c Assistant Speech and Hearing Therapist at APADA/ BA (Association of Parents and Friends of the Hearing Challenged of the State of Bahia). APADA- Associação de Pais e Amigos dos Deficientes Auditivos - Bahia

**Keywords:** children, prognosis, hearing loss, communication, etiology

## Abstract

Hearing represents the main source for acquisition of language and speaking skills in childhood. In the first months of life, the hearing impaired child is deprived of sound stimulation in the most important period of development, and consequently, might present emotional, social and linguistic disorders. Therefore, it is of utmost relevance to learn about the main etiological factors that cause the auditory damage to trace a reliable nosological profile, and to take the appropriate measures to prevent and guide the family on the repercussions of hearing impairment in childhood.

**Aim:**

To characterize the etiology profile of hearing impairment in a reference center for hearing impaired children and adolescents.

**Methodology:**

We performed interviews, speech and hearing screening and analyses of medical charts of 87 hearing impaired children that were part of Associação de Pais e Amigos dos Deficientes Auditivos do Estado da Bahia (APADA-BA), trying to define etiology, gender distribution, age at diagnosis, level of hearing loss, age at hearing aid fitting, and rehabilitation.

**Results:**

Among the 87 children and adolescents who had undergone speech and hearing screening, we select a sample of 53 subjects, whose parents had come for three sessions of anamnesis and assessment. The main responsible etiological factor for hearing loss in the evaluated population was maternal rubella, amounting to 32% of the cases of deafness, followed by pyogenic meningitis with 20%, idiopathic cause with 15%, prematurity with 9%, heredity (deaf father or mother) and neonatal jaundice, which also presented 6% incidence; chronic otitis media represented 4%, use of misoprostol in the gestation, measles, ototoxicity and mumps, each factor with 2%.

**Conclusion:**

The present study demonstrated the heterogeneity of factors that cause hearing impairment, and the two main causes (rubella and pyogenic meningitis) still present high incidence in the studied population. We believe that preventive measures must be taken, especially in the prophylaxis of maternal rubella and extended vaccination of neonates and infants against bacterial meningitis.

## INTRODUCTION

The ear is an organ that enables human beings to exert one of the noblest superior functions: communication. It is through language that we have been able to organize the universe, to understand the world around us, to comprehend our fellow human beings, to convey and examine thoughts and feelings of others, to interact with the environment and to acquire knowledge^1^.

Language is a highly complex process, as it relates to the elaboration and symbolization of human thought to allow communication between men. The ability to understand oral language must be considered one of the most important measurable aspects of human auditory function^2^.

In hearing impaired children, language acquisition and development process can be put in harm's way, should diagnosis and intervention not be done in a timely manner.

Research has also indicated the existence of a critical period that occurs within the first years of a child's life for speech acquisition. Lack of proper auditory stimulation in childhood may preclude the complete development and maturation of central auditory pathways^3^.

According to the WHO, 42 million people above the age of three bear some sort of moderate to profound hearing loss. Over 4% of the children considered to be at high risk are diagnosed with moderate to profound hearing loss^4^.

This paper aims at characterizing the etiological profile of hearing impairment at a reference center for the treatment of hearing challenged children and adolescents, so as to enable future prevention and care measures.

The identification of the main etiologic factors surrounding infantile deafness is an important diagnostic tool to allow the proper implementation of public health procedures and, consequently, of effective prevention measures and guidance to families in regards of hearing impairment.

## MATERIALS AND METHOD

The APADA - BA (Association of Parents and Friends of the Hearing Challenged of the State of Bahia) is a nonprofit organization that provides specialized support to deaf children and adolescents by means of psychopedagogy, speech and hearing therapy, psychology, and employment bureau services. It also counts on the support of a public secondary school that works within APADA premises where teachers, aside from the traditional curricula, teach Brazilian Sign Language (LIBRAS) to students.

A case series cross-sectional study was carried out. All selected children and adolescents were interviewed three times (one general interview and two evaluation sessions). The adopted screening protocol comprised interviews and speech and hearing evaluations, in which the main etiologic factors were analyzed by assessing the subjects’ prenatal history, their neuropsychomotor development, and test results brought in by their families and those already available from the subjects’ charts.

The items analyzed were distribution by gender, age of diagnosis, hearing loss severity, age of hearing aids introduction, and speech and hearing rehabilitation sessions attendance.

The project was submitted to the Research Ethics Committee of the Federal University of Bahia, at the Medical School Graduate Department, and was granted permit # 89/ 2004, knowledge area code 4.07, level D, Group III.

## RESULTS

**Chart 1:**
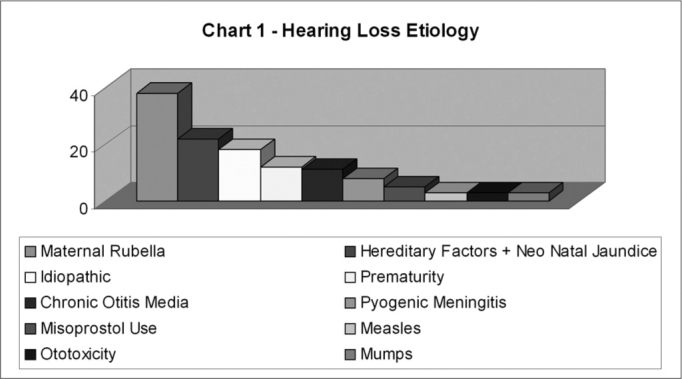
Diagnosis and Form of Communication.

Fifty-three subjects were chosen from the original sample of eighty-seven who underwent speech and hearing screening. Their parents attended all three interview and evaluation sessions. The main etiologic factor responsible for hearing impairment in the assessed population was mother's rubella, accounting for 32% of deafness cases, followed by pyogenic meningitis with 20%, idiopathic reasons with 15%, premature birth with 9%, inheritance (deaf father or mother) and neonatal jaundice with 6% each; chronic otitis media accounted for 4%, use of misoprostol during pregnancy, measles, ototoxicity, and mumps were also present, each with 2% (Chart 1).

**Chart 2:**
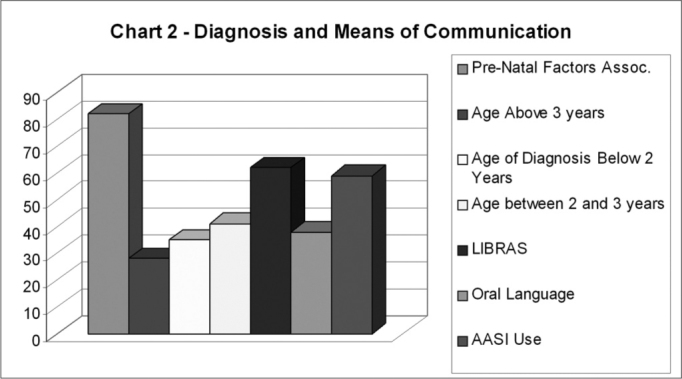
Hearing Impairment Etiologic Factors.

Thirty-one (58%) of the 53 subjects were males and 22 (42%) were females. Ages varied between 4 and 18 years. In the speech and hearing evaluation interview the main causes for hearing loss onset on the prenatal, perinatal, and postnatal periods were addressed, the age of diagnosis, the type of hearing aid device used, and the subjects’ ability to use either oral language or the Brazilian Sign Language. Results are described below (Chart 2).

Prenatal factors were identified in 43 of the 53 subjects in the sample (81%). Age of diagnosis < 2 years - 34%, between 2 and 3 years - 40%, and above 3 years of age - 26%. The hearing aid device found predominantly was AASI (58%), while 42% of the children never wore or discontinued the use of the device for various reasons (device broke, could not afford to buy batteries), and had not worn it in over 6 months.

The vast majority of the subjects used the Brazilian Sign Language as their main form of communication (62%), while only 38% used oral language as their principal means of communication.

**Chart 3:**
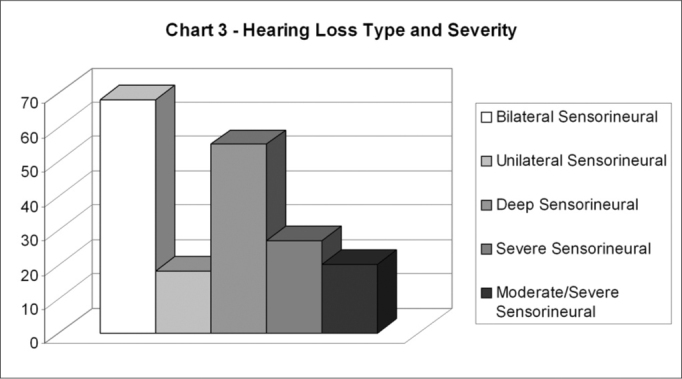
Type and Degree of Hearing Impairment.

As for the type of hearing impairment, the entire sample presented sensorineural hearing impairment and 33 individuals (62%) presented audiometric configuration compatible to bilateral loss, while only 10 children (18%) had unilateral loss. Thirty subjects in the sample (56%) had profound loss, 25% of them had severe loss, while 19% presented moderate to severe loss. (Chart 3)

## DISCUSSION

According to Walch et al.^5^, the data gathered from the subjects’ parents on the inheritance-related, prenatal, perinatal and postnatal causes for impairment onset is the most valuable information to establish disease etiology.

Infantile development depends basically on two aspects: the child's individual characteristics – organic and emotional conditions and environmental characteristics – social and familial conditions; and learning opportunities. The global development – cognitive, linguistic, and emotional – is determined by the interaction between these various factors^6^.

Inherited hearing impairment is considered to be of prenatal origin, and in this stage fetal infections play a role of paramount importance as causal factors. The main fetal infection to cause hearing impairment is rubella. However, with the introduction of immunization programs the incidence of this disease has decreased in developed countries in the last few decades. But rubella and other fetal infections are still quite prevalent in the Brazilian northeastern states, and should always be taken into consideration in the etiologic analysis of infantile congenital hearing loss^7^.

Later in childhood, the biggest cause of hearing impairment is meningitis, a disease that may lead to profound hearing loss. About one out of every one thousand newborns present hearing loss, and two out of every one thousand children begin to experience deafness within the first three years of life. These facts by themselves justify audiologic investigation in children not only when they are born, but also throughout their first years of life^8^.

The current trend in regards to hearing impairment points to diagnosis, etiology, and treatment within the boundaries of pediatric otological and otoneurological diseases, apart from neonatal and infant screening tests aiming at early hearing rehabilitation^9^.

One of the biggest challenges in pediatric audiology lies in the cases of hearing losses of unknown etiology in childhood. Parents must be thoroughly interviewed. Information coupled with data from audiologic tests can lead to the development of an etiologic diagnosis of the patient's hearing impairment. It is also recommended to include non-audiologic tests such as serology, imaging, ophthalmologic examination, and genetic evaluation. A broad interdisciplinary approach may offer valuable insight into uncovering unknown etiologies^10^.

Changes in the prevalence rates of infantile hearing impairment have been observed throughout the years. And many and different are the epidemiological aspects to be considered from country to country and even inside one same nation. In most developed countries the pediatric health care systems monitor children's development and offer follow-up and rehabilitation programs^11^.

Broad educational programs must be implemented to advise health care workers of the harm brought about by hearing impairment. The development of informational material can play a key role in the early detection of hearing impairment^12^.

## CONCLUSION

The etiologic identification done at the APADA population unveils many of the main factors at play in hearing impairment. It also calls for further studies in other centers so that a nosological profile of deafness can be properly outlined and input is provided to public health policy makers. The occurrence of diseases such as measles during pregnancy, and infectious contagious diseases such as meningitis, must be minimized so as to mitigate the impact of infantile hearing impairment.
